# Dynamics and Design Principles of a Basic Regulatory Architecture Controlling Metabolic Pathways

**DOI:** 10.1371/journal.pbio.0060146

**Published:** 2008-06-17

**Authors:** Chen-Shan Chin, Victor Chubukov, Emmitt R Jolly, Joe DeRisi, Hao Li

**Affiliations:** 1 Department of Biochemistry and Biophysics, University of California, San Francisco, San Francisco, California, United States of America; 2 Department of Pathology, University of California, San Francisco, San Francisco, California, United States of America; 3 Joint Graduate Group in Bioengineering, University of California, Berkeley, and University of California, San Francisco, San Francisco, California, United States of America; 4 Center for Theoretical Biology, Peking University, Beijing, China; Johns Hopkins University, United States of America

## Abstract

The dynamic features of a genetic network's response to environmental fluctuations represent essential functional specifications and thus may constrain the possible choices of network architecture and kinetic parameters. To explore the connection between dynamics and network design, we have analyzed a general regulatory architecture that is commonly found in many metabolic pathways. Such architecture is characterized by a dual control mechanism, with end product feedback inhibition and transcriptional regulation mediated by an intermediate metabolite. As a case study, we measured with high temporal resolution the induction profiles of the enzymes in the leucine biosynthetic pathway in response to leucine depletion, using an automated system for monitoring protein expression levels in single cells. All the genes in the pathway are known to be coregulated by the same transcription factors, but we observed drastically different dynamic responses for enzymes upstream and immediately downstream of the key control point—the intermediate metabolite α-isopropylmalate (αIPM), which couples metabolic activity to transcriptional regulation. Analysis based on genetic perturbations suggests that the observed dynamics are due to differential regulation by the leucine branch-specific transcription factor Leu3, and that the downstream enzymes are strictly controlled and highly expressed only when αIPM is available. These observations allow us to build a simplified mathematical model that accounts for the observed dynamics and can correctly predict the pathway's response to new perturbations. Our model also suggests that transient dynamics and steady state can be separately tuned and that the high induction levels of the downstream enzymes are necessary for fast leucine recovery. It is likely that principles emerging from this work can reveal how gene regulation has evolved to optimize performance in other metabolic pathways with similar architecture.

## Author Summary

Single-cell organisms must constantly adjust their gene expression programs to survive in a changing environment. Interactions between different molecules form a regulatory network to mediate these changes. While the network connections are often known, figuring out how the network responds dynamically by looking at a static picture of its structure presents a significant challenge. Measuring the response at a finer time scales could reveal the link between the network's function and its structure. The architecture of the system we studied in this work—the leucine biosynthesis pathway in yeast—is shared by other metabolic pathways: a metabolic intermediate binds to a transcription factor to activate the pathway genes, creating an intricate feedback structure that links metabolism with gene expression. We measured protein abundance at high temporal resolution for genes in this pathway in response to leucine depletion and studied the effects of various genetic perturbations on gene expression dynamics. Our measurements and theoretical modeling show that only the genes immediately downstream from the intermediate are highly regulated by the metabolite, a feature that is essential to fast recovery from leucine depletion. Since the architecture we studied is common, we believe that our work may lead to general principles governing the dynamics of gene expression in other metabolic pathways.

## Abstract 3

A quantitative, high-temporal resolution study of gene induction in a metabolic pathway reveals an intricate connection between the regulatory architecture and the dynamic response of the system, pointing to possible principles underlying the design of these pathways.

## Introduction

In the past several years, genomic approaches have dramatically accelerated the discovery of biological regulatory networks. Combined with detailed biochemical and genetic studies, these approaches have yielded the intricate wiring diagrams for many biological systems. Although revealing, such wiring diagrams are usually drawn as arrows representing activation or repression that link regulators with the genes they regulate, and typically, one can only make qualitative statements (such as whether a gene is activated or repressed) based on network architecture. Genetic and biochemical studies traditionally focus on gene regulation under steady growth conditions, and it is often difficult to rationalize the complex design of a regulatory system based only on these tasks.

To understand the functional significance and design principles of complex regulatory networks, it is essential to analyze the dynamical output quantitatively. In engineering systems such as feedback control, specifications for dynamics—such as speed of the response and settling time—strongly constrain the possible choices of network architecture and control parameters [1]. It is expected that the dynamic properties of the cellular response are important determinants for its fitness in an ever-changing environment. Consequently, certain features of the network architecture and parameters may be selected for their relevance to dynamics, as opposed to the steady-state behavior [2]. A number of recent studies have explored the connection between architecture, dynamics, and fitness; examples include rationalizing simple network motifs by their dynamical response properties [3,4], connecting just-in-time expression with fitness advantage [5], and justifying seemingly redundant regulatory mechanisms by their contributions to the different aspects of the dynamical response [6].

We explored the significance of dynamics in the regulation of metabolic pathways. Regulation of metabolic activity is of central importance for single cell organisms such as Escherichia coli and yeast, since they must respond to a constantly changing nutritional environment. Previous genetic and biochemical studies have elucidated the structure of various metabolic pathways and the associated regulatory circuits. Emerging from these studies is a basic architecture that regulates a linear branch of a biosynthetic pathway [7,8]. This architecture is characterized by a dual-feedback mechanism to control the metabolic flux and the synthesis of the enzymes in the pathway. The metabolic flux is generally controlled by end product feedback inhibition of the first enzyme unique to the pathway, and the expression of enzymes is regulated by transcription factors that can sense either the end product (e.g., tryptophan biosynthesis in E. coli [9]), or an intermediate (e.g., leucine biosynthesis in yeast). Transcriptional activation of a pathway by an intermediate is particularly widespread (e.g., lysine and adenine biosynthesis in yeast [10,11], lysine and methionine biosynthesis in *E. coli* [12,13]), which influenced our choice of the leucine pathway as a case study. Although all of these pathways have been studied extensively, so far data for the quantitative dynamical response have been scarce.

**Figure 1:**
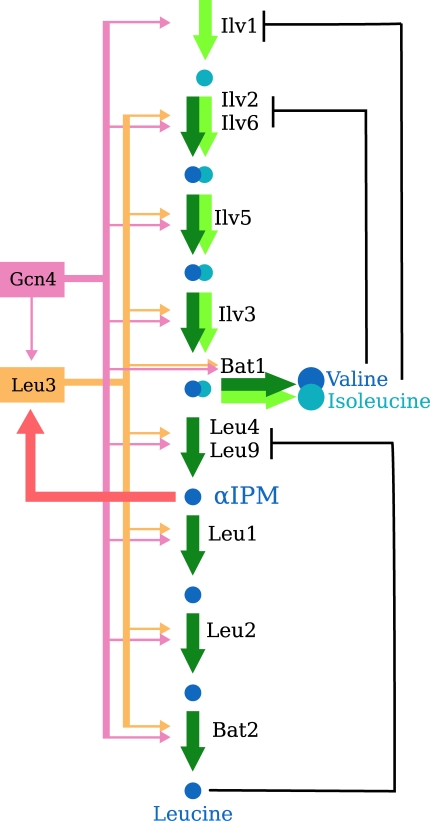
A Summary of the Leucine, Valine, and Isoleucine Biosynthesis Pathway in S. cerevisiae Enzymes are labeled next to their respective reactions, indicated by dark green arrows for the leucine and valine biosynthesis pathways, and light green for the isoleucine pathway. Metabolites are shown as blue circles, with the final end products and the key intermediate αIPM labeled on the figure. Allosteric regulation of enzymes by end products is shown by the black lines. The transcription factors Gcn4 and Leu3 are shown as boxes, with arrows to the genes displaying the fact that they are thought to potentially regulate all the genes in the pathway. Leu3 activity is dependent on αIPM level, as shown by the red arrow. For completeness, we include the arrow showing that Leu3 expression is thought to be Gcn4-dependent. See the text for further description on how the pathway is thought to be activated under starvation conditions.

The leucine biosynthetic pathway in the yeast *Saccharomyces cerevisiae* is summarized in Figure 1. This pathway converts pyruvate to leucine by the sequential reactions catalyzed by nine different enzymes. Part of the pathway is shared by valine biosynthesis, and several enzymes are also shared by the isoleucine biosynthetic pathway. There are three major regulatory features. First, leucine can bind to Leu4, inhibiting its catalytic activity [14]. Second, the branch-specific transcription factor Leu3 is known to be able to regulate the expression of all the genes in the pathway [15,16]. The activation domain of Leu3 is shielded when the pathway is inactive, and it is the binding of the metabolic intermediate α-isopropylmalate (αIPM) to Leu3 that unmasks its activation domain and allows it to activate the transcription of its targets. Finally, the pathway is also regulated by the transcription factor Gcn4, which is responsible for the general amino acid starvation response. Gcn4 controls a few hundred targets, including most of the genes in the leucine biosynthesis pathway, under amino acid starvation conditions [8,17]. It is known that combinatorial regulation by Gcn4 and branch-specific regulators such as Leu3 is a general scheme for controlling the synthesis of different amino acids. However, the effect of multiple regulators on the dynamics of gene expression remains uncharacterized.

## Results

### Monitoring Protein Abundance as a Function of Time Using an Automated System

To study gene induction with high accuracy and high temporal resolution, we built an automated system to monitor protein abundance in single cells. Although similar systems have been built before to acquire time-dependent population data [18–20], they have not been applied to systematic analysis of the dynamics of a genetic network. Our system consists of parallel batch cultures, in which yeast strains with different genes tagged by green fluorescent protein (GFP) are grown, connected to a flow cytometer by a syringe pump. Both sample delivery and data collection are controlled automatically by a computer with software written by the authors (see Figure S3 for a schematic of the design). The automated system we have built allows us to monitor up to six different strains simultaneously for several hours. Since a large number of cells (∼10^5^ to 10^6^) are sampled in short time intervals (1 to 7 min per sample), we obtain accurate and highly reproducible induction profiles. Previously, it had been shown in a genome-wide study that measuring protein abundance by GFP tagging and flow cytometry yields highly reproducible data and is less susceptible to experimental variation compared with other techniques such as Western blotting or mRNA microarray measurements [21].

**Figure 2:**
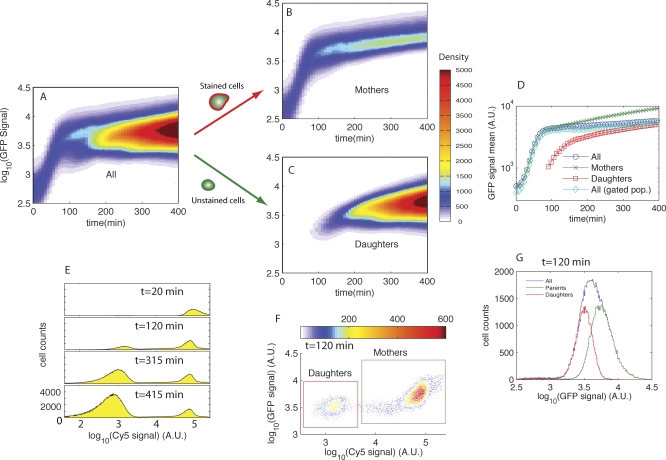
. Decomposition of the Time Course by Separating Parent and Daughter Cells (A) The time evolution of the histogram of protein abundance. Colors correspond to histogram height at each time point. The bimodal distribution can be decomposed into two simpler time courses for (B) the mother population and (C) the daughter population by using the cell wall dye. (D) Comparisons between the time courses obtained by decomposition of populations and by the general gating procedure using the FSC and SSC signals. The time course obtained by a gating procedure has an artificial drop due to the newborn cells. (E) The evolution of the histograms of the cell wall dye signal through four time points. The peaks on the right correspond to the mother cells and the peaks on the left correspond to the newborn cells. (F) The scatter plot shows the distribution of the GFP signal and the cell wall dye signal at 120 min. The color indicates the local cell number density. The mother and daughter cells show distinct distributions in both channels. (G) The separation of the bimodal GFP signal distribution into two individual distributions from mother and daughter cells using the information from the cell wall dye.

A major advantage of single cell measurement by flow cytometry is that it gives complete information on the distribution of protein abundance in a large population, instead of an average number obtained from a bulk measurement. This is particularly important when the cell population is inhomogeneous, where different cohorts might behave differently. Shown in Figure 2A is the time course of *LEU2-GFP* induction after switching from synthetic complete media (SCD) to synthetic media without leucine (SCD-Leu). Following media transfer, *LEU2* is induced and the distribution of *LEU2-GFP* levels in the population moves upward smoothly and gradually evolves into a bimodal shape. We find that such a bimodal distribution is due to continuous cell division. The population with the lower GFP level consists of the newly formed daughter cells, which receive less protein than the mother cells due to asymmetric cell division [22].

Despite the fact that many previous studies have used the population distribution of GFP level as a measure of gene expression, no satisfactory solution has been found for separating the effects due to the inhomogeneous population from those due to gene regulation at the single-cell level. The conventional approach is to sample cells with more uniform size, selected from within a narrow range close to the median of the forward-scattering channel (FSC) and the side-scattering channel (SSC). Nevertheless, this method would still show an artificial decrease in gene expression due to cell division (Figure 2D). To help resolve this problem, we use a dye to distinguish the newly formed daughter cells from the mother cells, in a procedure similar to that used by Porro and coworkers [23–26]. The cells are stained with Cy5 before inoculation. Since the cell wall of the newly formed cells is synthesized after inoculation, it carries few Cy5 dye molecules. By monitoring the GFP channel and the Cy5 channel simultaneously, we were able to decompose the overall distribution into the distributions of the mother and daughter subpopulations. Such decomposition allows us to accurately reconstruct the gene induction profile for the original mother subpopulation, which is more homogeneous and less sensitive to the effects of cell division (Figure 2) (see Materials and Methods for more details). As seen in Figure 2D, this approach eliminates the artifact of falling GFP levels, found either by looking at the whole population or by using conventional gating methods.

### Differential Response for Genes Upstream and Downstream of the Key Control Point αIPM

Using the system described above, we first measured the induction profiles of all the genes in the leucine biosynthesis pathway after transfer from SCD to SCD-Leu media. The high accuracy and high temporal resolution allow us to follow the change of the distribution and to calculate the rate of change of the GFP levels in the population (see Materials and Methods), which closely reflects the rate of protein production because the lifetimes of the GFP tagged proteins are much longer than the typical response time [27], whereas the maturation time is shorter [28].

**Figure 3:**
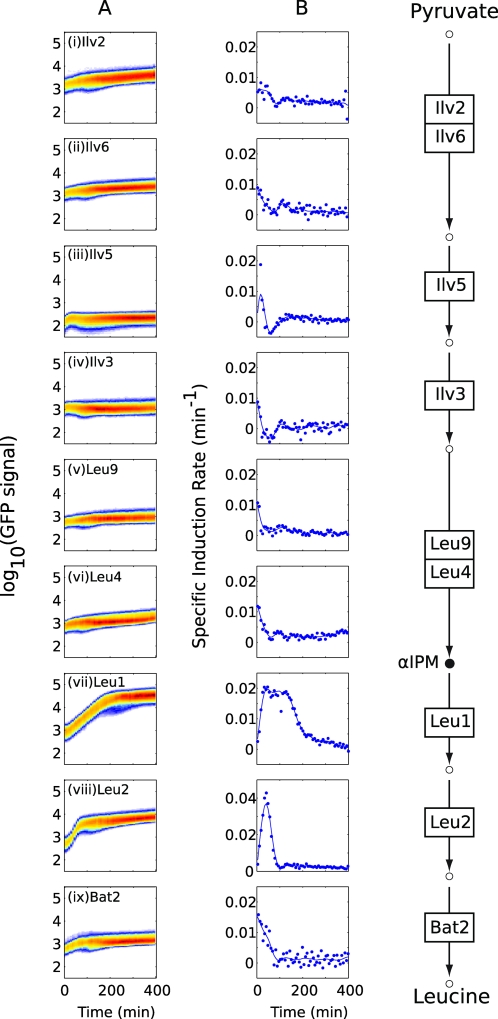
Measured Dynamic Profiles of all Pathway Genes (A) The measured time course of gene induction of all nine genes in the pathway ordered according to their positions in the pathway shown on the right. For each time point, we obtain a histogram of protein abundance using the observed normalized (background subtracted) GFP signal from the mother population. The color corresponds to histogram height at each time point. (B) The specific induction rate of each gene in the pathway during leucine starvation. The dots in the plot are the rates calculated using nearby time points. The solid curves are spline fits to guide eyes. The weakly induced enzymes (Ilv6, Ilv2, Ilv3, Ilv5, Leu4, Leu9, and Bat2) have high specific induction rate at the beginning and drop quickly. The two enzymes immediately downstream of αIPM, Leu1 and Leu2, have very distinct induction profiles from the other enzymes. They are also the most strongly induced genes in the pathway. The significant lag in the Leu1 curve compared to the Leu2 curve is likely due to a growth defect in the Leu1-tagged strain.

A striking feature we observed from these induction profiles is that the genes upstream of the intermediate αIPM (which serves as a key control point) and those that are immediately downstream display drastically different responses. The upstream genes (*ILV2*, *ILV6*, *ILV3*, *ILV5*, *LEU4*, and *LEU9*) were quickly induced but displayed a small-fold change ranging from 2- to 4-fold. The specific rates of change (defined as 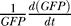 ) for these genes was at the maximum (about 1% per min) immediately following the transfer of media and then dropped to nearly zero in less than 50 min (Figure 3A and 3B, i–vi). In contrast, the two downstream genes (*LEU1* and *LEU2*) showed a relatively slower induction profile but much higher-fold changes. The rate of change for *LEU1* and *LEU2* both started low and reached their maximum (2% per min for *LEU1* and 5% per min for *LEU2*) in approximately 50 min. The overall fold changes for both genes after 400 min exceed 20-fold (Figure 3A and 3B, vii–viii). Interestingly, the third downstream gene, *BAT2*, whose product catalyzes the last step of leucine biosynthesis, displayed an induction profile similar to upstream enzymes, with a quick induction and an overall 2-fold change (Figure 3A and 3B, ix). Bat2 is a multi-functional enzyme shared by the valine and isoleucine biosynthetic pathways (see Figure 1).

### Distinct Dynamic Profiles Caused by Differential Regulation

To investigate the mechanism underlying the different responses for genes in the pathway, we measured their expression time courses under different genetic perturbations. Previous studies demonstrated that several genes in the leucine pathway are coregulated by the general regulator Gcn4 and the branch-specific transcription factor Leu3 [7]. Gcn4 is activated by general amino acid starvation and is controlled by translational regulation [29]. Under general amino acid starvation, uncharged tRNA activates the kinase Gcn2, which phosphorylates the translation initiation factor eIF2-α, leading to the translation of Gcn4. To analyze the relative role of the general signal (uncharged tRNA) through Gcn2/Gcn4 and the pathway specific signal (αIPM) through Leu3 in determining the dynamical response, we measured gene induction profiles in *LEU3* and *GCN2* knockout strains.

**Figure 4:**
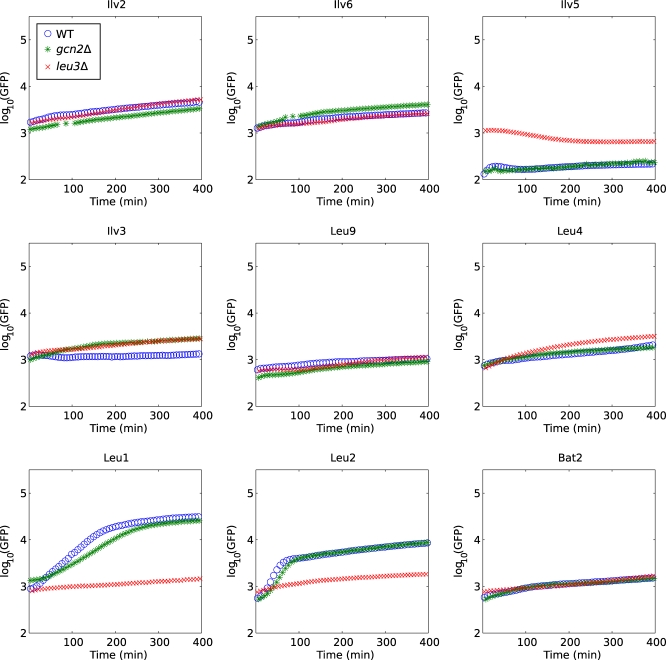
Comparisons of the Gene Induction Profiles in Wild-Type, *leu3*Δ, and *gcn2*Δ Backgrounds Shown are mean GFP levels following leucine depletion measured in three different backgrounds: wild type for blue circles, *leu3*Δ for red crosses, and *gcn2*Δ for green asterisks. While the deletion of *LEU3* completely abolishes the induction of Leu1 and Leu2, the effect on the upstream genes and on Bat2 is insignificant. Deleting *GCN2* also has relatively little effect compared to the *LEU3* deletion.

Figure 4 compares the detailed induction profiles of all the genes in the leucine pathway in wild-type background to the profiles of the same genes in *leu3*Δ and *gcn2*Δ background. We again observed qualitatively different behaviors for Leu1 and Leu2 in comparison with the rest of the pathway. The deletion of *LEU3* almost completely abolished the induction of these two downstream enzymes, making it clear that Leu3 is the major inducer of Leu1 and Leu2. The effect of *LEU3* deletion on the upstream enzymes and Bat2 is less pronounced. Leu4 and Ilv3 show a slight increase in gene expression level in the *leu3*Δ background. The other upstream enzymes, as well as Bat2, have dynamic profiles almost indistinguishable from wild type, except that the basal level of Ilv5 in *leu3*Δ background is much higher than that in the wild-type background. There are several possible explanations for the slight increase in expression of some of the upstream genes. As reported previously [30], Leu3 without αIPM can act as a repressor. It is therefore possible that in the wild type, Leu3 acts to repress upstream genes and these genes are derepressed in the *leu3*Δ strains. Another possibility is that in the *leu3* deletion strains, Leu1 and Leu2 are not expressed, which leads to a severe leucine deficit in SCD-Leu media, activating a general starvation response.

In contrast to the *LEU3* knockout, the *GCN2* deletion has a much less pronounced effect on either the upstream or downstream genes. The basic dynamic profiles of all genes are comparable to the wild-type strains. Since many studies have suggested that the upstream genes are inducible by Gcn4 under general amino acid starvation [8,29], this result suggests that Gcn2-dependent activation is weak under our experimental conditions, where only leucine is scarce in the medium. Most likely, the leucine-specific induction is efficient enough that the intracellular leucine concentration never drops to a sufficiently low level to activate the general amino acid starvation responses.

**Figure 5:**
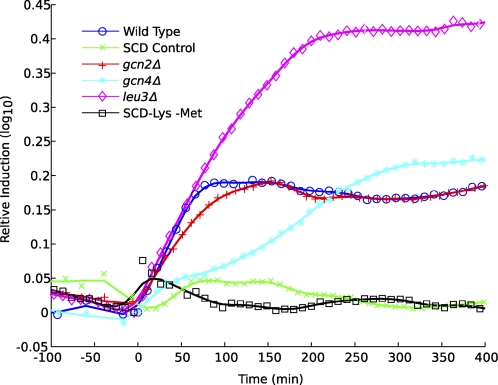
Comparisons of Induction Profiles of Leu4-GFP in Different Media and Genetic Backgrounds by Dilution and Continuous Sampling Cell cultures are grown in bioreactors containing SCD media, and monitored using the automated sampling system. At time *t* = 0, growing cell cultures with GFP-tagged Leu4 are diluted into fresh media. For the curves labeled “Wild Type,” “*gcn2*Δ,” “*leu3*Δ,”and “*gcn4*Δ,” the corresponding strains are diluted from SCD to SCD-Leu medium, decreasing by 12-fold the concentration of leucine in the environment. The curve labeled “SCD control” is a control in which wild-type cells are diluted into the same SCD medium, and the curve “SCD-Lys-Met” corresponds to dilution of wild type into SCD-Lys-Met medium, decreasing the concentrations of lysine and methionine by 12-fold, but keeping constant the concentration of leucine. The log_10_ GFP levels are normalized by subtracting the log_10_ basal level (defined by the level at *t* = 0).

To further analyze the initial fast induction dynamics of the upstream genes, we also used a different approach to quickly induce the pathway genes under a strictly controlled environment. Instead of subjecting the cells to spinning, washing, and staining, we simply diluted the SCD medium with SCD-Leu, lowering the concentration of leucine by about 10-fold in a few seconds. We continuously monitored the protein abundance before and after the dilution and observed that genes in the pathway are visibly induced within minutes. This approach eliminates the dead time (∼15 min) due to media transfer/staining. It also eliminates potential artifacts in gene induction due to possible stress caused by the media transfer/staining protocol. The dilution method allowed us to consistently detect small inductions (less than 20% change) within minutes after the change of environment. While it is no longer possible to separate mother and daughter populations with this method, that separation is most useful for the analysis of the later part of the time course when cell division effect becomes significant. Using the dilution method, we have analyzed the fast induction profiles of one of the upstream genes under different genetic and media perturbations. The resulting induction profiles for *LEU4* are shown in Figure 5. It is clear that the fast induction of *LEU4* is due to the depletion of leucine, since there are no observable changes in expression under the controls in which we add the same media or a media lacking lysine and methionine. Deletion of the branch-specific factor *LEU3* does not seem to affect the initial fast induction, since the induction curves for the wild-type and mutant strain are nearly identical for the first 70 min or so. The mutant strain eventually has higher induction, possibly due to a severe leucine deficit that triggers a stronger general starvation response. Deletion of *GCN2* leads to a minor but observable effect, resulting in a slightly slower induction profile. In contrast, deletion of *GCN4* has a much stronger effect, with the initial fast induction almost completely abolished. Taken together, these data suggests that the fast response of the upstream genes is specifically induced by leucine depletion, is independent of the branch-specific regulator Leu3, and that the general regulator Gcn4 is involved, possibly via a Gcn2-independent pathway. Our experimental observations lead to the following model for the dynamics of the pathway when the leucine level in the growth media is reduced. The response of the upstream genes consists of transient and minor inductions. This induction, combined with the release of Leu4 from leucine inhibition leads to a quick increase in αIPM synthesis. The accumulation of αIPM and its binding to the transcription factor Leu3 activates the sustainable and large amplitude expression of Leu1 and Leu2 to reinforce the branch specific response. The induction of the downstream genes leads to more effective conversion of αIPM to the end product and the rebalance of the intracellular leucine concentration.

### Modeling the Dynamics of Gene Induction and Intracellular Leucine Recovery

**Figure 6:**
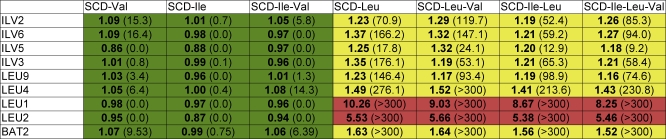
Gene Expression in Media Lacking Various Branched Chain Amino Acids Cells were grown in SCD medium, spun down, and switched to either one of seven test media, or SCD. Values are fold difference between mean GFP levels in specified medium and mean GFP levels in SCD after 320 min. Cells are color-coded by this value (*r*): green for *r* < 1.1, yellow for 1.1 < *r* < 5, and red for *r* > 5. Numbers in parentheses are –log_10_(*p*), with *p*-values calculated by a *T*-test between the SCD population and the test medium population.

To test whether the above intuitive picture captures the essential features of the response, we built a simplified mathematical model to quantitatively describe the observed dynamics and make new predictions. To minimize the number of parameters and simplify the model, we make the assumption that the enzymes not specific to the leucine pathway (Ilv2, −3, −5, −6, and Bat2) are not rate limiting, i.e., they are operating far below *V*_max._ There are several justifications for this—first, none of these genes are induced in media lacking isoleucine and valine even though they comprise part of the corresponding biosynthetic pathway (Figure 6). Furthermore, their induction is transient and an order of magnitude lower than that of Leu1 and Leu2 in media lacking leucine. These observations suggest that the change of the flux through these enzymes is mainly due to the change of the metabolite concentration. A simple calculation indicates that the flux through each of these enzymes can quickly balance the flux upstream of the enzyme in a time much shorter than the typical pathway response time (see Text S2). As such, we will ignore these enzymes in our kinetic model. In our model, the pathway response is dominated by the release of inhibition on the upstream enzyme Leu4 and the transcriptional up-regulation of the downstream enzymes Leu1 and Leu2. Assuming that the metabolite upstream of Leu4 is always abundant, the equations describing the essential dynamics of the pathway can be written as:

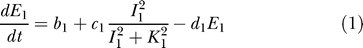


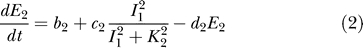


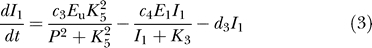


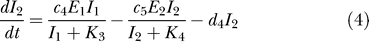


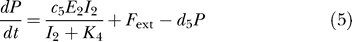



**Figure 7:**
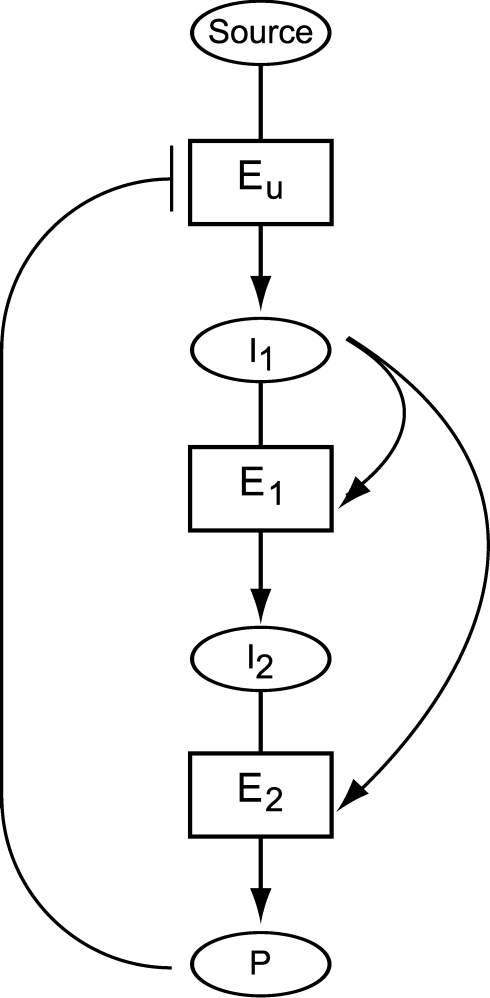
A Schematic Diagram of the Mathematical Model The correspondence to biological entities is as follows: *E*_u_ represents Leu4 and Leu9, *E*_1_ represents Leu1, *E*_2_ represents Leu2, *I*_1_ represents αIPM, *I*_2_ represents βIPM, and *P* represents leucine. The regulatory relationships included in the model are the feedback inhibition of Leu4 and Leu9 by leucine, and the transcriptional up-regulation of Leu1 and Leu2 by αIPM (through Leu3).

A schematic of the model is shown in Figure 7. The model consists of a set of differential equations describing the dynamics of the two intermediates *I*_1_ (αIPM) and *I*_2_ (product of Leu1, β-isopropylmalate, abbreviated as βIPM); the downstream enzymes *E*_1_ and *E*_2_, representing Leu1 and Leu2 respectively; the upstream enzyme *E*_u_, representing Leu4 (treated as constant); and the end product *P* (leucine). The rates of protein production (the first two terms in Equations 1 and 2) are assumed to be proportional to the mRNA level. Since the half-lives of the mRNAs of the downstream genes are short (∼10 to 20 min [31]), we simply assume that the mRNA level is proportional to the rate of transcription, which has a low basal level (*b*_1_ and *b*_2_ terms) in the absence of αIPM and a much higher level when αIPM is present (*c*_1_ and *c*_2_ terms). It is known that Leu3 is constitutively bound to its DNA binding sites [32]. We thus assume that the transcriptional induction by αIPM is proportional to the probability that αIPM is bound to the preformed Leu3–DNA complex, modeled by a sigmoid function of the αIPM concentration. The dynamics for the intermediates (Equations 3 and 4) are governed by the standard Michaelis-Menten kinetics. The activity of the upstream gene is controlled by the feedback inhibition by the end product (first term in Equation 3). The dynamics of the end product are determined by the rate of synthesis and the rate of usage (*d*_5_ *P*), which is assumed to be proportional to the leucine concentration. The *F*_ext_ term reflects the external leucine flux. It is presumed to be positive in the rich media condition, and set to zero when leucine is missing in the environment. The remaining terms (*d*_1–4_) are used to model dilution effects due to cell growth (see Materials and Methods for details).

### Modeling the Downstream Gene Induction

**Figure 8:**
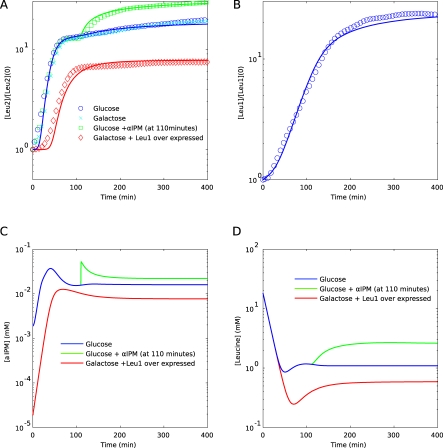
Overview of Time Course Data and Model Predictions (A) Comparison of Leu2 induction under different perturbations. The induction curves are almost identical when the cells grow in different media, glucose (blue circles) and galactose (cyan crosses). The green squares show the measured time course when external αIPM was added at 110 min. The red diamonds indicate the time course of the Leu1 over-expressed strain. The data shown are concentrations, calculated by estimating cell volume from scattering data (see Materials and Methods for details). The solid lines with corresponding colors show the fitting results using our model. (B) Comparison of Leu1 time course data to the model. Symbols indicate experimental measurement results while the solid line shows the fitting results using our model. Incorporating only the slow growth rate into the model is sufficient to produce the slower induction observed for Leu1-GFP (see Materials and Methods for details). (C) The time courses of the concentration of the metabolic intermediate αIPM inferred by our model. (D) The time courses of the concentration of intracellular leucine inferred by our model.

We first tested whether the model can reproduce the quantitative dynamics we observed for the downstream enzymes. By adjusting the free parameters in Equations 1–5 within specified bounds (based on previous knowledge and physical estimates, see Materials and Methods for details), the model is capable of producing downstream enzyme induction profiles (*E*_1_ and *E*_2_ in our model) that fit the observed Leu1 and Leu2 induction profiles (Figure 8A and 8B). The Leu1 profile is produced by taking into account the slower growth of the Leu1-GFP strain, which is done by scaling the dilution and usage terms by the relative growth rate. Given the fitting of the downstream enzyme levels, the model also predicts the dynamics of the intermediate αIPM and the intracellular leucine level (Figure 8C and 8D), which are difficult to measure with high temporal resolution. In particular, the model predicts that the αIPM concentration starts at a low level, reaches its maximum around 50 min, and then decreases to the new steady-state level. The intracellular leucine level gets depleted with a characteristic time of ∼15 min, reaches minimum around 50 min, and then recovers to steady state after a period of overshooting (Figure 8D).

The fitting of the gene expression profiles puts constraints on the choice of the parameters but does not yield a unique solution. Similar quality fitting can be achieved by different sets of parameters. Part of this parameter degeneracy is intrinsic to the nonlinear model, which exhibits “soft modes” in the parameter space, where the output depends only on certain combinations of the parameters [33]. While the exact parameter values cannot be uniquely inferred from our model and experimental data, there are robust features of the dynamics that are independent of the choice of the parameters, which gives our model predictive power.

### Predicting the Dynamic Responses to New Perturbations

To test the predictive power of the model, we considered several environmental and genetic perturbations and used the model to predict how these perturbations would alter the pathway response. We then compared the predicted induction profiles to those measured by experiment. Because αIPM is the key control point of the pathway, we considered two different perturbations that would affect the dynamics of αIPM concentration. For the first perturbation, we increased the external flux of αIPM by adding exogenous αIPM to the media at a specific time after the transfer from SCD to SCD-Leu media. This is modeled by the addition of a constant external flux term φ_ext_ in Equation 3. The second is a genetic perturbation in which we constitutively overexpress the enzyme Leu1. This is modeled by the addition of a constant term *b*_ext_ in Equation 1, where *b*_ext_ represents the constant production of additional Leu1 due to overexpression. As one would expect, the model predicts that increasing the αIPM flux leads to increased induction of Leu2. In contrast, overexpressing Leu1 leads to more efficient depletion of αIPM, leading to decreased Leu2 induction. The predicted induction profiles for several different values of φ_ext_ and *b*_ext_ are plotted in Figure S1.

We performed both perturbations experimentally. In the αIPM addition experiment, we started with two identically prepared cell cultures in two different reactors, added 7.5 mM αIPM into one of the reactors 110 min after inoculation, and monitored the induction profiles of the two cultures simultaneously. The two induction profiles were nearly identical before the addition of αIPM, and started to deviate from each other in less than 10 min after the addition of αIPM. As expected, the addition of αIPM leads to higher induction of Leu2. By allowing the parameter φ_ext_ to vary (representing the fact that we do not know exactly how much αIPM from the medium is absorbed by the cell) we can fit the induction profile accurately (Figure 8A, green lines). The remaining parameters are fixed from fitting the wild-type data. Notice that while we have to fit one additional parameter, the parameter is sufficient to fit the timing, amplitude, and shape of the extra induction due to the addition of αIPM.

The Leu1 overexpression experiment was performed by transfecting the wild-type cell with a plasmid containing a galactose-inducible *GAL1*/*GAL10* promoter driving Leu1 expression. When galactose is used as the carbon source, this strain expresses Leu1 at a high level constitutively. As predicted by our model, Leu2 expression was not as high as in wild type, and again, by fitting only *b*_ext_ (the extra Leu1 produced by the *GAL1*/*GAL10* promoter), we can reproduce the temporal profile (Figure 8A, red lines). Again, the model correctly predicts the effect of the perturbation.

### Dynamics of the Intracellular Leucine Recovery and the Features of the Pathway Design

We have observed contrasting dynamical responses for enzymes upstream and downstream of the control point αIPM and showed that the differential dynamics are caused by differential regulation by Leu3. In addition, we have observed that the enzymes immediately downstream of the intermediate have high-fold induction. Some of these features are also present in other metabolic pathways controlled by a similar regulatory architecture (e.g., Lys9 in lysine biosynthesis and Ade17 in adenine biosynthesis (VC, CC, and HL, unpublished data)). Are these features of gene expression linked to the dynamics of the system's recovery?

To address this question, we explored the connection between gene expression and the intracellular leucine level, which we assume to be a limiting factor for the recovery of cell growth. Using the mathematical model, we analyzed how changing properties of the network may affect the dynamics of gene expression and intracellular leucine recovery.

We first explored the connection between basal expression of the downstream enzymes and leucine dynamics. Our model predicts that elevating the basal expression level of Leu1 (by overexpression) reduces αIPM levels and consequently Leu2 expression, as confirmed by the experiments. The model also predicts that constitutively overexpressing Leu1 leads to a significant delay in the intracellular leucine recovery (Figure 8D, red line), since Leu2 becomes rate limiting. A possible solution is to express both downstream enzymes at high levels constitutively. However, this strategy is not optimal when leucine is abundant in the environment, and the enzymes are not needed.

**Figure 9:**
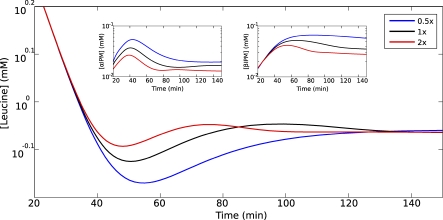
The Leucine Concentration When the Promoter Strengths in the Model (*c*_1_ and *c*_2_ in Equation 1) are Changed From the Values Fitting the Experimental Data (1x) The insets show αIPM and βIPM concentrations. Increasing *c*_1_ and *c*_2_ (2x, red line) leads to a faster leucine recovery, whereas decreasing *c*_1_ and *c*_2_ (0.5x, blue line) delays recovery. Neither change affects the steady state concentration.

A better strategy for improving the dynamics of the response is to tune the strength of the induction (fold change) for the downstream enzymes, instead of increasing the basal expression level. This may speed up the recovery of intracellular leucine during the transition to leucine-poor media while minimizing the cost in leucine-rich media. We implemented this perturbation to the system by changing the parameters *c*_1_ and *c*_2_ in our model, which correspond to the rates of transcription from the *LEU1* and *LEU2* promoters when bound by the Leu3-αIPM complex. Figure 9 shows that when the induction rates of the downstream enzymes are increased, the model predicts that the speed of leucine recovery is improved.

Interestingly, although changing the kinetic parameters modifies the dynamic response, the leucine concentration in the steady state remains the same. This turns out to be a general property of the model as a consequence of flux conservation, as long as the effective upstream enzyme level only depends on the end product and the dilution due to cell growth can be neglected (which is generally true based on the model parameters we inferred, see Text S1). These observations lead us to speculate that the regulatory architecture of the system makes it possible to separately tune steady state and dynamics, and that certain features are chosen for their influence on efficient dynamics, even though they do not contribute to the maintenance of steady-state nutrient level.

## Discussion

We have analyzed the dynamics of a regulatory module that controls the leucine biosynthetic pathway in yeast, and explored the connection between dynamics and network architecture. Using an automated system for monitoring protein expression level in single cells, we have systematically measured the changes in expression level for the genes in the pathway quantitatively, with high temporal resolution. Compared to past studies—e.g., RNA expression based on microarray experiments [34]—we can distinguish the temporal expression profiles of enzymes on the same pathway with much higher accuracy. Our approach observes features that are not seen when the network is at steady state and can provide a key to rationalizing aspects of the regulation that may not be necessary for maintaining growth in a constant environment.

One remarkable feature we observed is the differential response (both in terms of amplitude and timing) of the enzymes in the pathway. For enzymes shared by the isoleucine and valine pathways (Ilv2, −6, −5, −3, and Bat2), the induction is fast and transient with a small amplitude. For the four enzymes specific to the leucine branch (Leu4, −9, −1, and −2), we observed two different responses separated by the key control point: the intermediate αIPM, which couples metabolic feedback to transcriptional regulation. While the two enzymes upstream of the control point (Leu4 and Leu9) displayed a fast transient induction with small amplitude, the two enzymes downstream of the control point (Leu1 and Leu2) showed a slow but sustained induction with large amplitudes. These differential responses are caused by differential regulation by the branch-specific regulator Leu3 and the general regulator Gcn4: Leu1 and Leu2 are strictly controlled by Leu3, thus their activation requires the accumulation of αIPM, which is the slow step; the fast transient induction of other enzymes seems to be dependent on Gcn4 but not Leu3. These observations are in accord with the previous genetic analysis that showed that all the enzymes in the pathway, except Leu1 and Leu2, can be induced by the general amino acid starvation response [8], and that basal expression of Leu2 is suppressed by a DNA-bound but inactive Leu3 [35]. However, the vastly different effect of Leu3 on upstream and downstream genes and its consequence on the kinetics of gene induction had not been explored before.

Mechanistically, it is unclear how Leu3 acts differently at the promoters of the upstream and downstream genes. We speculate that the different regulation might be achieved by the positioning of the Leu3 binding site in the promoter, and in particular its positioning relative to the Gcn4 binding site. We have observed a correlation between the response profiles and the binding site arrangement. At the *LEU1* and *LEU2* promoters, the Leu3 binding sites are close to the transcription start site, whereas the Gcn4 binding sites are further upstream. At most of the other promoters, the Gcn4 sites are closer to the transcription start site.

Why are there two types of qualitatively different regulation and consequently different dynamical responses? For the enzymes shared by valine and isoleucine pathways, it can be rationalized that they should not be strictly controlled by the branch-specific regulator Leu3, since the cell needs to turn on valine and isoleucine synthesis in environments lacking valine and isoleucine but with leucine abundant (which keeps Leu3 inactive). From this perspective, the leucine biosynthetic pathway provides an interesting model system to investigate cross regulation of pathways that share a subset of enzymes. Our preliminary study with media lacking all possible combinations of the three amino acids indicates that all the enzymes of the pathway adjust their expression level only in response to leucine depletion (Figure 6). Thus with the depletion of valine and/or isoleucine but not leucine, the metabolic flux is turned on by the release of end product inhibition without changing the enzyme levels. This suggests that these shared enzymes are operating far below saturation in rich media and that their induction is not essential for leucine production when leucine is depleted. The small transient induction probably serves to fine-tune the speed and the magnitude of the response.

The more intriguing observation is that even the four enzymes specific to the leucine pathway (Leu4/Leu9, Leu1, and Leu2) display different responses, depending on whether they are upstream or downstream of the control intermediate αIPM. Could the separation of two different responses by αIPM be a coincidence? If αIPM acts as a positive regulator on the pathway that regulates both production and consumption of itself, the feedback effects are different between upstream genes and downstream genes. For the downstream genes, increased expression leads to more effective conversion of αIPM to βIPM, thus the feedback is negative. In contrast, for the upstream genes, increasing expression leads to more synthesis of αIPM, thus the feedback is positive. The different feedback effects suggest that the observed differential regulation and differential dynamics for genes separated by αIPM may not be a coincidence, but rather a consequence of natural selection that optimizes the performance of the system.

Can Leu4/9 be strictly controlled by Leu3 (just like Leu1 and Leu2) and expressed only when the pathway needs to be turned on? We argue that such a hypothetical design would lead to negative consequences on the speed as well as the stability of the system. In the real system where Leu4/9 are constitutively expressed and their activities controlled by leucine feedback inhibition, the depletion of leucine can quickly lead to αIPM synthesis. In the hypothetical design, the accumulation of αIPM needs activation of Leu4/9, and the activation of Leu4/9 in turn needs accumulation of αIPM, thus the kinetics of turning on can be slow. Moreover, a strong induction of the upstream enzymes implies strong positive feedback, and an overly strong positive feedback would lead to unnecessary overproduction of both upstream enzymes and the controlling metabolite αIPM itself, possibly leading to uncontrolled activation of the pathway.

The regulation of the leucine biosynthetic pathway has the extra complexity that the enzymes upstream and downstream of αIPM are located in different cellular compartments: the former in the mitochondria and the latter in the cytoplasm [7]. However it is unlikely this feature is responsible for the distinct dynamic profiles we have observed—for instance, Bat2 is located in the cytoplasm along with Leu1 and Leu2 but has a dynamic profile more similar to the upstream enzymes. Nevertheless, compartmentalization and intracellular transport can add an additional layer to the regulation, which needs to be explored further.

As we have shown in our experimental observation and theoretical modeling, the dramatic induction of the downstream enzymes dominates the leucine synthesis dynamics under our experimental conditions. Together with the end-product inhibition of Leu4 by leucine, the high induction of the downstream enzymes Leu1 and Leu2 by αIPM is arranged so as to satisfy the following requirements: (1) reduce the unnecessary enzyme production when cells are growing in rich media and (2) boost Leu1 and Leu2 production in such a way as to minimize the delay in growth due to lack of intracellular leucine. The release from end-product inhibition ensures a kick-start mechanism to provide the intermediate metabolites. To complete the synthesis, the downstream enzyme can either be expressed at a high level constitutively or be produced only when the intermediate metabolite is produced. The first choice is disadvantageous because resources are wasted. The second mechanism would need an extra regulation step, which must be efficient to minimize the delay in growth. Previous studies show that Leu3 is expressed and bound to the promoter regions of *LEU1* and *LEU2* constitutively and activated by αIPM. The fact that growth rate is unaffected under leucine depletion (Figure S2) suggests that this system has evolved to optimize its response, resulting in efficient production of leucine.

Transcriptional regulation by the uncharged tRNA/Gcn2/Gcn4 pathway does not seem to be a major factor in our experimental conditions, since none of the *gcn2*Δ strains show expression profiles that are significantly different from wild type under leucine depletion. One likely explanation for this is that leucine synthesis is turned on so efficiently by the branch-specific mechanism that the intracellular leucine level is never sufficiently depleted to turn on the uncharged tRNA pathway. Some evidence for this comes from the aforementioned fact that cell growth is not significantly affected by the switch from SCD to SCD-Leu media (Figure S2), while cell growth is typically stunted under more severe conditions that are known to activate the pathway, for instance, starvation conditions where synthesis of one or more amino acids is also significantly impaired. We did observe leucine-specific induction of upstream enzymes, which does not strongly depend on either Leu3 or Gcn2, but depends on Gcn4, pointing to the interesting possibility of a leucine-specific induction of Gcn4 via a Gcn2- independent pathway.

The systematic and quantitative data we obtained has made it possible to build and test a simplified mathematical model. We show that the model captures the essential features of the regulation, since it can quantitatively account for the observed dynamics as well as predict the pathway's response to new perturbations. The model also makes a number of interesting predictions that connect dynamics with network design. One prediction of the model is that certain observed features such as the high-fold induction of the enzymes immediately downstream of the control point are required for efficient dynamics but not for maintaining the steady-state leucine level, thus the steady state level and the speed of the response can be separately tuned. We have observed that the enzyme immediately downstream of the regulatory intermediate metabolite has the highest-fold induction across several other pathways with similar architectures suggesting that this may be a general design feature evolved to optimize the dynamical response.

The basic dynamic features we observed in the leucine biosynthetic pathway in yeast are different from the just-in-time dynamics previously reported for amino acid biosynthesis pathways in E. coli [5]. Zaslaver et al. reported just-in-time dynamics characterized by the following features: the closer the enzyme is to the beginning of the pathway, the faster the response and the higher the fold induction. In our experiments, although we observed differences between upstream and downstream genes, we did not observe timing differences among the upstream enzymes or among the downstream enzymes. What seems to matter is whether the enzyme is upstream or downstream of the control point, and whether the enzyme is specific to the leucine pathway. Furthermore, the downstream enzymes have higher-fold induction, which seems to be opposite to the trend observed in some of the pathways in E. coli. However, the pathways for which strong just-in-time trends are observed are not controlled by the same regulatory architecture. This certainly suggests that network architecture can play a significant role in the evolution of gene regulation, since different architectures can impose different constraints and demands on the dynamics of gene expression.

Understanding the structure-function relationship of biological regulatory networks is a big challenge. The dynamic features of a network are important components of its function and have been largely ignored in traditional genetic and biochemical analysis. The network architecture we studied in the leucine biosynthesis pathway is widely repeated for regulating many other biosynthetic pathways. Our experimental observation and theoretical modeling may lead to new understanding of how these metabolic pathways are regulated and the principles that have evolved to optimize their performance. As the structures of more and more regulatory systems are elucidated, it will be possible to compare the architecture and quantitative dynamics across different systems and in different species. Without question, such studies will shed light on the functional constraints and general design principles of biological regulatory systems.

## Materials and Methods

### Strains.

All yeast strains used in this research are derived from DBY7286 (MATa ura3–52). The C-terminal GFP-tagged strains were constructed using the plasmid pFA6a-GFP(S65T)-KanMX6 as previously described [36] with modifications. For most enzymes in the pathway, we found that the GFP-tagged enzyme strains have no observable growth phenotype with the exception of GFP-tagged Ilv5 and Leu1 strains. In those two strains, we found that that the cell cultures grow slower compared to other tagged and wild-type strains. It is likely that the enzymatic function is affected by the GFP tagging. Nevertheless, we found that the cells are still viable in SCD-Leu media.

The LEU3 knockout strains were constructed by replacing the LEU3 locus with the URA3 gene from Kluyveromyces lactis by one-step homologous recombination.

The strain over-expressing Leu1p was constructed in the following manner: *LEU1* was amplified by PCR from genomic DNA (yeast strain SK1) with forward primer oEJ636, containing an Xho1 restriction site, and reverse primer oEJ637 containing a SacII restriction site. The PCR product containing *LEU1* was subcloned into the pCR2.1 vector (Invitrogen) and sequenced to make plasmid pEJ663. The XhoI/ SacII fragment of plasmid pEJ667, containing *LEU1,* was further subcloned into pRS426 vector, containing promoter GAL1-LacZ (pEHB22,073, graciously donated by the Blackburn lab), and analyzed by sequence analysis to make pEJCS001, containing the *LEU1* gene driven by the *GAL1* promoter. Plasmid pEJCS001 was transformed into yeast cells. *LEU1* expression was induced on YPG media after overnight growth on raffinose media.

The strains used in this research are listed in Table S1.

### Automated flow cytometry measurement system.

We designed a customized system for sampling cells. Cells growing in bioreactors controlled by Sixfors laboratory fermenter (Appropriate Technical Resources) are automatically delivered to a flow cytometer (LSR II, BD Biosciences) through a syringe pump system. The system was constructed with an automatically controlled pump base (PSD/3, Hamilton Company), an eight-port valve (HVCX 8–5, Hamilton Company) and a 250-μl syringe. The schematic plot of the setup is shown in Figure S3. The pump is controlled by software written by the authors using Borland C++ 4.0. The software monitors and decodes the raw data stream sent from the flow cytometer to synchronize the sample delivery and the fluorescence readings. Extra washing steps are programmed between samples to reduce sample cross contamination below 1%. For each time point, 25 μl of sample is injected into the flow cytometer at a flow rate of 25 μl per minute.

### Growth conditions and measurements of gene induction.

For the time courses for which mother-daughter separation was done, the following protocol was used: overnight cell cultures diluted to OD_600_ 0.05–0.1 were grown to mid-log phase (OD_600_ 0.3∼0.5) in 100-ml flasks. The cells were spun down and washed with phosphate buffered saline (PBS) and stained with ¼ pack Cy5 dye (Cy5 Post-Labeling Reactive Dye Pack, Amersham Biosciences) in 250 μl PBS for 10 min. After staining, cells were washed again, and then inoculated into the bioreactors. The typical starting cell density ranged from OD_600_ 0.05–0.1. We found that the variation of the initial cell density did not affect the induction time courses for the first 6 h. The bioreactors were preheated to 30 °C and stirred at 240 rpm. No clumping of cells was observed from the flow cytometer readings.

For the dilution time courses, overnight cultures were diluted to OD_600_ = 0.01–0.03 and grown to mid-log phase (OD_600_ 0.05–0.1) They were then inoculated into bioreactors containing SCD media, and further grown to about OD_600_ of about 0.4. Gene expression was monitored using the automated system during this time. At *t* = 0, 30 ml of cell culture was diluted into 330 ml of SCD-Leu (or other, see Figure 5) media, with negligible disruption in sampling.

For the measurements in media with different combinations of branched chain amino acids (results shown in Figure 6), overnight cultures were diluted to OD_600_ of approximately 0.05 into 96-well plates containing SCD media. They were grown for 4 h, and spun down. SCD medium was discarded, and cells were quickly reinoculated into the test media.

The fluorescence reading is saved in the standard FCS 3.0 format. The GFP fluorescence is measured using the area of the FITC channel (510 nm) and the signal of cell staining is measured in APC (650 nm). The measured GFP signal is calibrated for autofluorescence using the average GFP signal from the measurement of a wild-type non-GFP–tagged strain as a reference. Then the corrected GFP signals for each cell are binned to get their distributions. Instead of using mean or median values from gated populations, which are sensitive to the shape of the distributions, we directly compared the distributions from two consecutive measured samples. For two consecutively measured samples, we calculate the amount shift of GFP signal from the stained populations in log-scale to best overlap the distributions (details in the Text S3). This shift gives the specific induction rate 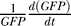 . We find this method is more robust against artifacts due to the small changes in the shape of the distributions and does not depend on arbitrary gating cutoffs.

### Modeling the dynamics of the pathway.

To compare our experimental data, which is in units of GFP per cell, with the model, which is formulated in units of concentration, we implemented a procedure to estimate cell volume from our flow cytometry data. We use FSCA (forward scattering area) to the 4/3 power as an approximate measure of volume, as this method gives a good correlation between volume and GFP level for individual timepoints. This procedure determines the concentration up to a constant. The constant is fixed by assuming an average cell volume of 50 μm^3^ and a conversion of 10 protein molecules per GFP unit.

In an effort to constrain the parameters in the model as much as possible, we performed a comprehensive literature search for measurements of the chemical constants that correspond to parameters in our model. We were able to find measurements, mostly in vitro, of the constants corresponding to *c*_3_, *c*_4_, *c*_5_, *k*_1_, *k*_2_, *k*_3_, *k*_4_, *k*_5_ and *P*(0) [37–42] (see Table S2 for values). Since these measurements do not always correspond perfectly to in vivo values, we allowed these parameters to change by 10-fold (1 order of magnitude) up or down during the fitting. A similar procedure was followed for the other free parameters. *d*_5_ was estimated by a rough calculation of the amount of leucine that must be used during each cell cycle, whereas *c*_1_ and *c*_2_ were estimated by finding the maximal values of the Leu1and Leu2 specific induction rates, and assuming that each promoter was approximately half-activated at this time. These estimates were also allowed to vary within a factor of 10. The dilution parameters *d*_1_, *d*_2_, *d*_3_, and *d*_4_ are estimated as ln(2)/*T*, where *T* is the observed doubling time (approximately 120 min). Since these parameters should correspond fairly accurately to our measured data, we allow them to vary only 2-fold from the estimate. *E*_1_(0) and *E*_2_(0) are taken from the basal level in our measurements and were not allowed to vary. Finally *b*_1_, *b*_2_, *I*_1_(0), *I*_2_(0), and *F*_ext_ are determined by the other parameters by enforcing the condition that the system is at steady state at *t* = 0.

Since the Leu1-GFP strain grows significantly slower than the wild-type or other tagged strains, we had to account for this effect in our model. This was done by multiplying the dilution and usage terms by a constant corresponding to the ratio of doubling times between the Leu1-GFP and Leu2-GFP strains. This constant was not allowed to vary in the fitting.

The set of ordinary differential equations was numerically integrated, and the fit to the data computed by a simple least-square method. The error was minimized by a simulated annealing algorithm, using a Metropolis Markov Chain Monte Carlo method [43,44]. The algorithm performed a thorough search of the parameter space, and found several classes of solutions that are able to fit the data almost equally well. At this point, several additional criteria based on physical intuition were used to filter the solutions: we required that the basal transcription terms (*b*_1_ and *b*_2_) be larger than the αIPM-dependent transcription terms at *t* = 0. We also required that the maximum αIPM value be of the same order of magnitude as *k*_1_ and *k*_2_. This narrowed the solution space to only a few distinct solutions, of which we chose the one that minimized the fitting error. In general, the results presented in Figure 9, as well as the qualitative profiles of the leucine and αIPM curves in Figure 8, are not dependent on the particular choice of solution.

## Supporting Information

Figure S1 Model Predictions of a Range of Experimental Perturbations (A) Predictions of the Leu2 time courses under the perturbations by changing the exogenous αIPM flux (φ_ext_) at 110 min.
(B) Prediction of the Leu2 time courses under the perturbations of Leu1 overexpression (*b*_ext_) at different level.
(214 KB EPS)

Figure S2 Comparison of the Growth Curve for Cells Growing in Synthetic Complete Media without Leucine (SCD-Leu) and in Synthetic Complete Media (SCD) The numbers of cells are measured by counting the number of events from flow cytometer measurements. The red line indicates the slope for growth at a doubling time equal to 111 min.
(267 KB PNG)

Figure S3 The Schematic Plot for the Setup of the Automatic Measurement System (1.2 MB TIF)

Figure S4 A Graphical Display of the Calculation of the Specific Induction Rate (503 KB PNG)

Table S1 Strains Used in This Research (12 KB PDF)

Table S2 Estimates for the Parameters in the Model and the Final Fitted Values (13 KB PDF)

Text S1 The Steady State Intracellular Leucine Level Is Independent of Most Model Parameters (29 KB PDF)

Text S2 The Upstream Fluxes Can Balance Much More Quickly Than the Pathway Response Time (36 KB PDF)

Text S3 Details on the Method Used to Align Consecutive Histograms (24 KB PDF)

## Abbreviations

αIPM: α-isopropylmalate
GFP: green fluorescent protein
SCD: synthetic complete media
