# Supplementary material for: Dynamics and Design Principles of a Basic Regulatory Architecture Controlling Metabolic Pathways
Source: PLoS Biol. 2008 Jun 17;6(6):e146. doi: 10.1371/journal.pbio.0060146 (PMC2429954; doi:10.1371/journal.pbio.0060146)
Supplement: Supplementary file 1 [file pbio.0060146.sd001.pdf]

**Supplementary Note 1 : The steady state intracellular leucine level is independent of most model parameters.**

To see why the leucine level at steady state depends only on the upstream flux in our model, consider the following equations. which determine the steady state leucine value:

$$\frac{dI_1}{dt} = \frac{c_3 E_u K_5^2}{P^2+K_5^2} - \frac{c_4 E_1 I_1}{I_1+K_3} - d_3 I_1$$

$$\frac{dI_2}{dt} = \frac{c_4 E_1 I_1}{I_1+K_3} - \frac{c_5 E_2 I_2}{I_2+K_4} - d_4 I_2$$

$$\frac{dP}{dt} = \frac{c_5 E_2 I_2}{I_2+K_4} + F_{ext} - d_5 P$$

Assuming the decay terms for the intermediates ($d_3 I_1$ and $d_4 I_2$) can be neglected, the flux terms for the intermediates cancel in the summation of these three equations, which leads to

$$\frac{c_3 E_u}{P^2+K_5^2} + F_{ext} - d_5 P = 0$$

It follows that the solution for $P$ depends only on the upstream enzyme $E_u$ and the external flux $F_{ext}$, and not on the levels of the downstream enzymes.
