# Supplementary material for: Dynamics and Design Principles of a Basic Regulatory Architecture Controlling Metabolic Pathways
Source: PLoS Biol. 2008 Jun 17;6(6):e146. doi: 10.1371/journal.pbio.0060146 (PMC2429954; doi:10.1371/journal.pbio.0060146)
Supplement: Supplementary file 2 [file pbio.0060146.sd002.pdf]

Supplementary Note 2 : The upstream fluxes can balance much more quickly than the pathway response time.

Consider an enzyme E operating far below V_{max} in a linear pathway. Assume that the flux upstream of the enzyme changed at time t=0. We estimate how long it takes for the flux through the enzyme to balance the upstream flux. The dynamics of the substrate S of the enzyme, produced by the upstream flux, are governed by the equation

$$\frac{dS}{dt}=F_u-\frac{K_{cal}ES}{S+K_m} ,$$

where F_u is the upstream flux, and the second term is the flux through the enzyme given by standard Michaelis-Menten kinetics. Under the assumption that the enzyme is operating far below V_{max}, that is, $S<<K_m$, the equation reduces to

$$\frac{dS}{dt}=F_u-\frac{S}{\tau}$$, with the time constant $\tau=\frac{K_m}{K_{cal}E}$.

The solution to this equation is

$$\frac{S}{\tau}=F_u+(S_0-F_u)\exp(-t/\tau) ,$$

where S_0 is the initial concentration of the substrate. Thus the flux through the enzyme relaxes to the upstream flux F_u exponentially with time constant τ. To get an order of magnitude estimate of τ, we use K_m = 0.5 mM, $E = 10^4$ molecules/cell, which translates to about 0.3 μM assuming the cell volume is 50μm^3, and K_{cal} = 600/min. These parameters yield τ = 3 minutes. Thus the time it takes to reach flux balance is much faster than the typical response time of the pathway.
