# Supplementary material for: Dynamics and Design Principles of a Basic Regulatory Architecture Controlling Metabolic Pathways
Source: PLoS Biol. 2008 Jun 17;6(6):e146. doi: 10.1371/journal.pbio.0060146 (PMC2429954; doi:10.1371/journal.pbio.0060146)
Supplement: Supplementary file 3 [file pbio.0060146.sd003.pdf]

Supplementary Note 3 : Details on the method used to align consecutive histograms.

Figure S4: A graphical display of the calculation of the specific induction rate.

We use the population distributions from two consecutive samples measured at t_1 and t_2 to estimate the specific induction rate. The above figure shows the distributions of GFP signal (FITC) of two consecutive samples in log-scale. The distributions are approximately similar to log-normal distributions. Furthermore, simply shifting one of the distributions in log-scale typically can collapse two consecutive normalized distributions. This implies the rate change of protein abundances, $\frac{dP}{dt}$, is proportional to *P*, namely, the specific induction rate, defined as $r=\frac{1}{P}\frac{dP}{dt}$, is approximately a constant between two consecutive measured samples. As shown in the above figure, such an approximation indeed agrees with the observed data of the mother population.

The specific induction rate at $t=\frac{(t_1+t_2)}{2}$ is then calculated by determining the amount of shift that minimizes the difference between the collapsed distributions (by numerical optimization). To further reduce noise, we calculate the optimal shifting using the cumulative distribution function (CDF), which is smoother than the probability distribution function.
