# Supplementary material for: Dynamics and Design Principles of a Basic Regulatory Architecture Controlling Metabolic Pathways
Source: PLoS Biol. 2008 Jun 17;6(6):e146. doi: 10.1371/journal.pbio.0060146 (PMC2429954; doi:10.1371/journal.pbio.0060146)
Supplement: Supplementary file 4 [file pbio.0060146.st001.pdf]

Strain ID	Genotype (MAT**a**)
yCS0001	ura3-52 LEU1::GFP-KanMX
yCS0003	ura3-52 LEU2::GFP-KanMX
yCS0005	ura3-52 LEU4::GFP-KanMX
yCS0010	ura3-52 ILV3::GFP-KanMX
yCS0011	ura3-52 ILV6::GFP-KanMX
yCS0012	ura3-52 LEU9::GFP-KanMX
yCS0013	ura3-52 ILV5::GFP-KanMX
yCS0014	ura3-52 ILV2::GFP-KanMX
yCS0015	ura3-52 LEU1::GFP-KanMX leu3Δ::URA3
yCS0016	ura3-52 LEU2::GFP-KanMX leu3Δ::URA3
yCS0017	ura3-52 LEU4::GFP-KanMX leu3Δ::URA3
yCS0018	ura3-52 LEU9::GFP-KanMX leu3Δ::URA3
yCS0019	ura3-52 ILV6::GFP-KanMX leu3Δ::URA3
yCS0020	ura3-52 ILV3::GFP-KanMX leu3Δ::URA3
yCS0021	ura3-52 ILV5::GFP-KanMX leu3Δ::URA3
yCS0032	ura3-52 ILV2::GFP-KanMX leu3Δ::URA3
yCS0026	ura3-52 LEU1::GFP-KanMX gcn2Δ::URA3
yCS0027	ura3-52 LEU2::GFP-KanMX gcn2Δ::URA3
yCS0028	ura3-52 LEU4::GFP-KanMX gcn2Δ::URA3
yCS0028	ura3-52 LEU9::GFP-KanMX gcn2Δ::URA3
yCS0033	ura3-52 ILV2::GFP-KanMX gcn2Δ::URA3
yCS0034	ura3-52 IVL3::GFP-KanMX gcn2Δ::URA3
yCS0035	ura3-52 ILV6::GFP-KanMX gcn2Δ::URA3
yCS0037	ura3-52 IVL5::GFP-KanMX gcn2Δ::URA3
yCS0024	ura3-52 LEU1::GFP-KanMX P_{GAL1}-LEU1-URA3
yVC0003	ura3-52 LEU4::GFP-KanMX gcn4Δ::NAT
