# Supplementary material for: Dynamics and Design Principles of a Basic Regulatory Architecture Controlling Metabolic Pathways
Source: PLoS Biol. 2008 Jun 17;6(6):e146. doi: 10.1371/journal.pbio.0060146 (PMC2429954; doi:10.1371/journal.pbio.0060146)
Supplement: Supplementary file 5 [file pbio.0060146.st002.pdf]

Parameter	Estimate	Value
b_1	--	1.02×10^{-6} mM min^{-1}
c_1	1.7×10^{-4} mM min^{-1}	5.08×10^{-4} mM min^{-1}
d_1	0.0058 min^{-1}	0.0079 min^{-1}
K_1	0.25 mM	0.053 mM
b_2	--	3.5×10^{-7} mM min^{-1}
c_2	1.1×10^{-4} mM min^{-1}	5.18×10^{-5} mM min^{-1}
d_2	0.0058 min^{-1}	0.0064 min^{-1}
K_2	0.25 mM	0.035 mM
c_3E_u	2 mM min^{-1}	0.32 mM min^{-1}
c_4	500 min^{-1}	2.64×10^{3} min^{-1}
d_3	0.0058 min^{-1}	0.011 min^{-1}
K_3	0.5 mM	2.71 mM
c_5	800 min^{-1}	108 min^{-1}
d_4	0.0058 min^{-1}	0.0080 min^{-1}
K_4	0.3 mM	0.22 mM
d_5	0.3 min^{-1}	0.077 min^{-1}
K_5	0.1 mM	0.66 mM
F_{ext}	--	1.42 mM min^{-1} if $t<0$; 0 if $t>0$
$E_1(0)$	2.2×10^{-4} mM	2.2×10^{-4} mM
$E_2(0)$	7.9×10^{-5} mM	7.9×10^{-5} mM
$I_1(0)$	--	1.8×10^{-3} mM
$I_2(0)$	--	8.6×10^{-3} mM
$P(0)$	2 mM	18.4 mM
φ_{ext}	--	0.18 mM min^{-1}
b_{ext}	--	1.8×10^{-4} mM min^{-1}
